# Retrospective Chart Review of Patients with Self-Harm Seen as Liaison Psychiatry in Pakistan

**DOI:** 10.1192/j.eurpsy.2022.647

**Published:** 2022-09-01

**Authors:** M.F.A. Malik, B. Najeeb

**Affiliations:** Rawalpindi Medical University, Institute Of Psychiatry, Rawalpindi, Pakistan

**Keywords:** pakistan, liaison, self-harm

## Abstract

**Introduction:**

Self‑harm is an ‘act of self‑poisoning or self‑injury carried out by a person, irrespective of their motivation’. A history of self-harm is linked with suicide risk. A study in Pakistan found self-harm to be more common in young people with unemployment and interpersonal difficulties as common triggers. Expanding liaison psychiatry services leads to an earlier assessment of patients with self-harm.

**Objectives:**

To study the demographic and clinical variables of patients along with methods and precipitating factors of self-harm.

**Methods:**

A retrospective chart review of patients presenting with self-harm seen as a part of liaison psychiatry from October 2018 to June 2021.

**Results:**

A total of 168 cases were seen of which 10 were excluded due to incomplete data. Of 158 cases gender split was roughly in the middle, with 49.4% males (n=78) and 50.6% females (n=80). The mean age of patients was 27.59 with a range of 12-70, 40.5% belonged to the age group of 20-29 (n=64) (Figure 1).

**Figure:**
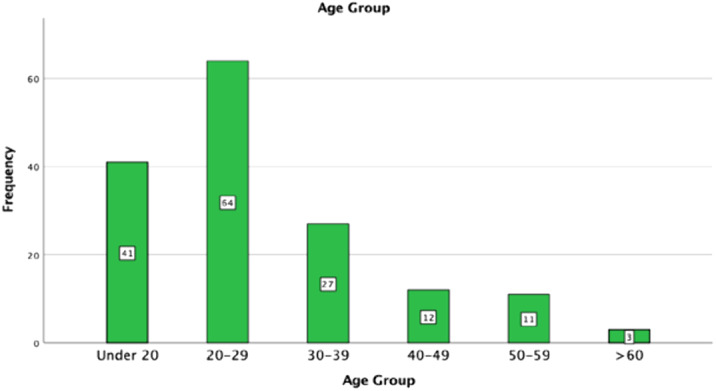

77.8% (n=123) had a past psychiatric history. The most common being depressive disorder 31.6% (n=50) and borderline personality disorder 30.4% (n=48). 35.4% of patients reportedly had previous attempts of self-harm. The most common methods being the use of sharp objects, rat-pill poisoning, and corrosive intake (Figure 2).

**Figure:**
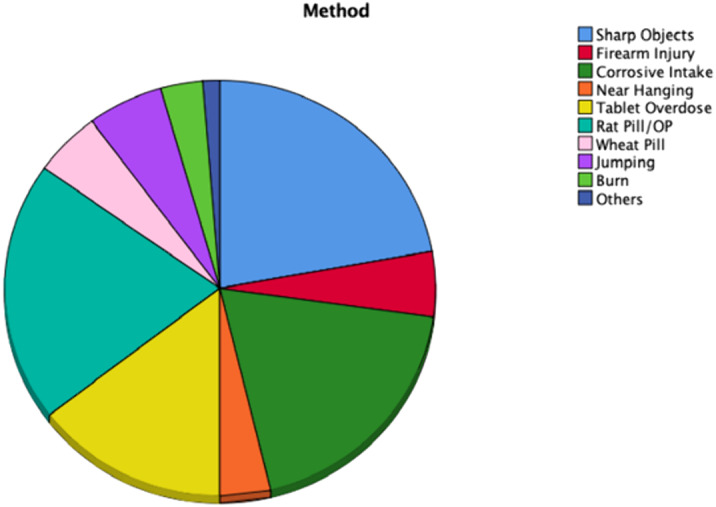

Conflicts with family and relationship difficulties were reported to be the most common precipitating factors.

**Conclusions:**

Self-harm is a challenging and frequent presentation. Patients may present with diverse characteristics and varying needs. Hence physicians must be prepared for timely liaison and prompt management.

**Disclosure:**

No significant relationships.

